# *N*-Cyclo­propyl-*N*-[2-(2,4-difluoro­phen­yl)-2-hy­droxy-1-(1*H*-1,2,4-triazol-1-yl)prop­yl]-2-(5-methyl-2,4-dioxo-1,2,3,4-tetra­hydro­pyrimidin-1-yl)acetamide dichloro­methane 0.62-solvate

**DOI:** 10.1107/S1600536811034295

**Published:** 2011-08-27

**Authors:** Nan Wang, Huan-Mei Guo, Gui-Ge Hou, Xin-Yue Hu, Qing-Guo Meng

**Affiliations:** a School of Pharmacy, Yantai University, Yantai 264005, People’s Republic of China; b Microscale Science Institute, Weifang University, Weifang 261041, People’s Republic of China; c School of Pharmacy, Binzhou Medical College, Yantai 264003, People’s Republic of China

## Abstract

In the title compound, C_21_H_22_F_2_N_6_O_4_·0.62CH_2_Cl_2_, the difluoro-substituted benzene ring forms dihedral angles of 54.6 (3)° with the mean plane of the thymine ring and 50.9 (2)° with the triazole ring. The dihedral angle between the thymine and triazole rings is 7.4 (3)°. In the crystal, inter­molecular N—H⋯N and O—H⋯O hydrogen bonds link the main mol­ecules into chains along [10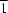]. The CH_2_Cl_2_ solvent mol­ecule was refined as partial occupancy over two sets of sites with refined occupancies of 0.308 (9) and 0.310 (8).

## Related literature

For the applications of azole and triazole compounds as anti­fungal agents, see: Singh (2001 ▶); Richardson (2005 ▶); Hobson (2003 ▶); Slavin *et al.* (2002 ▶); Wingard & Leather (2004 ▶); Fridkin & Jarvis (1996 ▶); Gallis *et al.* (1990 ▶); Sheehan *et al.* (1999 ▶); Denning (2002 ▶); Aoyama *et al.* (1984 ▶); Lamb *et al.* (1999 ▶).
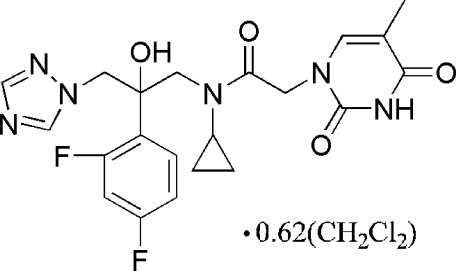


## Experimental

### 

#### Crystal data

C_21_H_22_F_2_N_6_O_4_·0.62CH_2_Cl_2_
*M* *_r_* = 512.93
Triclinic, 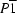

*a* = 8.474 (2) Å
*b* = 12.464 (3) Å
*c* = 13.410 (4) Å
α = 62.942 (4)°
β = 73.187 (4)°
γ = 83.746 (4)°
*V* = 1207.1 (6) Å^3^
*Z* = 2
Mo *K*α radiation
μ = 0.24 mm^−1^
*T* = 298 K
0.30 × 0.16 × 0.14 mm

#### Data collection

Bruker SMART APEX diffractometer
6058 measured reflections
4162 independent reflections
3053 reflections with *I* > 2σ(*I*)
*R*_int_ = 0.022

#### Refinement

*R*[*F* ^2^ > 2σ(*F* ^2^)] = 0.092
*wR*(*F* ^2^) = 0.209
*S* = 1.16
4162 reflections
356 parameters
42 restraints
H-atom parameters constrained
Δρ_max_ = 0.50 e Å^−3^
Δρ_min_ = −0.34 e Å^−3^

### 

Data collection: *SMART* (Bruker, 1997 ▶); cell refinement: *SAINT* (Bruker, 1997 ▶); data reduction: *SAINT*; program(s) used to solve structure: *SHELXS97* (Sheldrick, 2008 ▶); program(s) used to refine structure: *SHELXL97* (Sheldrick, 2008 ▶); molecular graphics: *SHELXTL* (Sheldrick, 2008 ▶); software used to prepare material for publication: *SHELXTL*.

## Supplementary Material

Crystal structure: contains datablock(s) global, I. DOI: 10.1107/S1600536811034295/lh5302sup1.cif

Structure factors: contains datablock(s) I. DOI: 10.1107/S1600536811034295/lh5302Isup2.hkl

Supplementary material file. DOI: 10.1107/S1600536811034295/lh5302Isup3.cml

Additional supplementary materials:  crystallographic information; 3D view; checkCIF report

## Figures and Tables

**Table 1:** Hydrogen-bond geometry (Å, °)

*D*—H⋯*A*	*D*—H	H⋯*A*	*D*⋯*A*	*D*—H⋯*A*
N6—H6⋯N2^i^	0.80	2.11	2.893 (5)	167
O1—H1*A*⋯O2^ii^	0.82	2.13	2.903 (4)	158

Symmetry codes: (i) 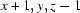; (ii) 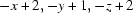.

**Fig. 1.**
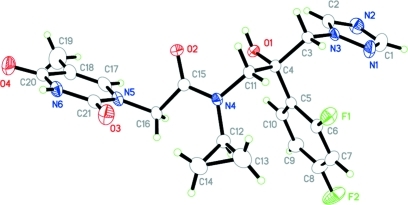
The molecular structure of the title compound showing displacement ellipsoids with 30% probability. The solvent molecule is not shown.

**Fig. 2.**
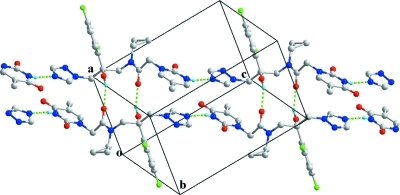
Part of the crystal structure of the title compound. Dashed lines indicate hydrogen bonds. Only H atoms involved in hydrogen bonds are shown. The solvent molecules are not shown.

## supplementary crystallographic information

### Comment

In recent years, fungal infections are prevalent diseases from which a large proportion of the human population suffers (Singh, 2001; Richardson, 2005; Hobson, 2003; Slavin *et al.*, 2002). The increased emergence of both superficial and systemic fungal infections has led to the massive increase in the rate of mortality, especially in immunocompromised individuals i.e. those suffering from tuberculosis, cancer or AIDS (Wingard & Leather, 2004; Fridkin & Jarvis, 1996). There are very few antifungal agents that can be used for life-threatening fungal infections. Several clinical drugs, such as amphotericin *B*,5-fluorocytosine, azoles (such as fluconazole and itraconazole) and echinocandins (such as caspofungin and micafungin) have been developed to reduce the impact of fungal diseases (Gallis *et al.*, 1990; Sheehan *et al.*, 1999; Denning, 2002). Among those, azoles, especially triazole antifungal agents, are used widely and efficiently. For over a decade, azoles have been a mainstay of the antifungal armamentarium. They act by competitive inhibition of the lanosterol 1410 which is the key enzyme in sterol biosynthesis of fungi (Aoyama *et al.*, 1984). Selective inhibition of CYP51 would cause depletion of ergosterol and accumulation of lanosterol and other 14-methyl sterols resulting in the growth inhibition of fungal cells (Lamb *et al.*, 1999). We have attempted to prepare some potential antifungal agents with improved activity and broader spectrum, by sythesizing a series of 1-(1*H*-1,2,4-triazole-1-yl)-(2,4-difluorophenyl)-3-[*N*-n-alkyl-*N*-(1-thyminyl)acetyl]-2-propanol compounds. Herein, we report the crystal structure of the title compound (I).

The molecular structure of the title compound is shown in Fig. 1. The difluoro-substituted benzene ring forms dihedral angles of 54.6 (3)° with the mean plane of the thymine ring and 50.9 (2)° with the triazole ring. The dihedral angle between the thymine and triazole rings is 7.4 (3)°. In the crystal, intermolecular N—H···N and O—H···O hydrogen bonds link the main molecules into one-dimensional chains (Fig. 2) along [101].

### Experimental

The synthesis of thymin-1-yl acetic acid: To a solution of thymine (30.00 g, 237.89 mmol) in water (150 ml) was added potassium hydroxide (51.25 g, 903.79 mmol). The solution was heated to 313 K, and then bromoacetic acid (49.58 g, 356.83 mmol) was added dropwise. The resulting solution was allowed to stir for 2 h at the same temperature. The solution was then brought to pH 5 with 36% HCl at 273 K. The white precipitate was filtered off and discarded. Then the filtrate was brought to pH 1 with 36% HCl. Solid was filtered off and dried and obtained in 83.5% yield, m.p 528–530 K.

The synthesis of the title compound: To a solution of 1-(1H-1,2,4-triazole)-2,2-[oxiranyl-(2,4-difluorophenyl)]ethane mesylate (16.75 g, 50 mmol) in ethanol (240 ml) was added triethylamine (16 ml, 110 mmol) and cyclopropylamine (6.93 ml, 100 mmol). The resulting solution was raised to 318 K and stirred for 10 h at the same temperature. Once concentrated to light yellow oil under vacuum, the residue was taken off with ethyl acetate and was treated with 1 M HCl. The aqueous phase was then brought to pH 8 with sodium carbonate and extracted with ethyl acetate. The organic phase was washed with water and brine, dried over anhydrous sodium sulfate, filtered, and concentrated under reduced pressure. A light yellow oil was obtained in 67.6% yield. A solution of the title compound (9.2 mg,0.02 mmol) in dichloromethane and methanol (4 ml, 1:1, *v*/*v*) was kept at room temperature. Upon slow evaporation of the solvent over about 10 d, colourless block-shaped crystals suitable for X-ray measurements were obtained.

### Refinement

All H atoms were placed in idealized positions and treated as riding,with C—H = 0.97 (CH_2_), 0.96 (CH_3_), 0.93 Å (CH_aromatic_), 0.98 Å (CH_tertiary alkyl_), N—H = 0.80 Å, O—H = 0.82 Å, and *U*_iso_(H) = 1.2*U*_eq_(N, CH and CH_2_), *U*_iso_(H) = 1.5*U*_eq_(OH and CH_3_). The CH_2_Cl_2_ solvent molecule was refined as partial occupancy over two sets of sites with refined occupancies of 0.308 (9) and 0.310 (8). The precision of this structure is lower than normal and this may be the result of the presence of the disordered solvent in the lattice.

### Crystal data

**Table:**

C_21_H_22_F_2_N_6_O_4_·0.62CH_2_Cl_2_	*Z* = 2
*M**_r_* = 512.93	*F*(000) = 532.2
Triclinic, *P*1	*D*_x_ = 1.411 Mg m^−^^3^
Hall symbol: -P 1	Mo *K*α radiation, λ = 0.71073 Å
*a* = 8.474 (2) Å	Cell parameters from 1529 reflections
*b* = 12.464 (3) Å	θ = 2.5–24.6°
*c* = 13.410 (4) Å	µ = 0.24 mm^−^^1^
α = 62.942 (4)°	*T* = 298 K
β = 73.187 (4)°	Block, colourless
γ = 83.746 (4)°	0.30 × 0.16 × 0.14 mm
*V* = 1207.1 (6) Å^3^	

### Data collection

**Table:**

Bruker SMART APEX diffractometer	3053 reflections with *I* > 2σ(*I*)
Radiation source: fine-focus sealed tube	*R*_int_ = 0.022
graphite	θ_max_ = 25.0°, θ_min_ = 1.8°
φ and ω scans	*h* = −10→8
6058 measured reflections	*k* = −14→11
4162 independent reflections	*l* = −15→10

### Refinement

**Table:**

Refinement on *F*^2^	Primary atom site location: structure-invariant direct methods
Least-squares matrix: full	Secondary atom site location: difference Fourier map
*R*[*F*^2^ > 2σ(*F*^2^)] = 0.092	Hydrogen site location: inferred from neighbouring sites
*wR*(*F*^2^) = 0.209	H-atom parameters constrained
*S* = 1.16	*w* = 1/[σ^2^(*F*_o_^2^) + (0.0687*P*)^2^ + 1.6778*P*] where *P* = (*F*_o_^2^ + 2*F*_c_^2^)/3
4162 reflections	(Δ/σ)_max_ < 0.001
356 parameters	Δρ_max_ = 0.50 e Å^−^^3^
42 restraints	Δρ_min_ = −0.34 e Å^−^^3^

### Special details

**Table:**

Geometry. All e.s.d.'s (except the e.s.d. in the dihedral angle between two l.s. planes) are estimated using the full covariance matrix. The cell e.s.d.'s are taken into account individually in the estimation of e.s.d.'s in distances, angles and torsion angles; correlations between e.s.d.'s in cell parameters are only used when they are defined by crystal symmetry. An approximate (isotropic) treatment of cell e.s.d.'s is used for estimating e.s.d.'s involving l.s. planes.
Refinement. Refinement of *F*^2^ against ALL reflections. The weighted *R*-factor *wR* and goodness of fit *S* are based on *F*^2^, conventional *R*-factors *R* are based on *F*, with *F* set to zero for negative *F*^2^. The threshold expression of *F*^2^ > σ(*F*^2^) is used only for calculating *R*-factors(gt) *etc*. and is not relevant to the choice of reflections for refinement. *R*-factors based on *F*^2^ are statistically about twice as large as those based on *F*, and *R*-factors based on ALL data will be even larger.

### Fractional atomic coordinates and isotropic or equivalent isotropic displacement parameters (Å^2^)

**Table:**

	*x*	*y*	*z*	*U*_iso_*/*U*_eq_	Occ. (<1)
C1	0.4568 (7)	0.7140 (5)	1.3061 (5)	0.0602 (15)	
H1	0.3778	0.7522	1.3425	0.072*	
C2	0.5856 (6)	0.5787 (4)	1.2724 (4)	0.0416 (11)	
H2	0.6213	0.5044	1.2752	0.050*	
C3	0.7947 (5)	0.7116 (4)	1.0885 (3)	0.0320 (10)	
H3A	0.8887	0.6649	1.1106	0.038*	
H3B	0.8256	0.7963	1.0532	0.038*	
C4	0.7508 (5)	0.6834 (4)	0.9990 (4)	0.0296 (9)	
C5	0.5887 (5)	0.7388 (4)	0.9760 (3)	0.0291 (9)	
C6	0.5620 (5)	0.8613 (4)	0.9364 (4)	0.0331 (10)	
C7	0.4170 (6)	0.9142 (4)	0.9172 (4)	0.0481 (12)	
H7	0.4040	0.9968	0.8914	0.058*	
C8	0.2910 (6)	0.8396 (5)	0.9378 (5)	0.0537 (14)	
C9	0.3077 (6)	0.7182 (4)	0.9764 (4)	0.0476 (12)	
H9	0.2210	0.6695	0.9894	0.057*	
C10	0.4563 (5)	0.6694 (4)	0.9955 (4)	0.0349 (10)	
H10	0.4680	0.5866	1.0227	0.042*	
C11	0.8951 (5)	0.7280 (4)	0.8885 (4)	0.0333 (10)	
H11A	0.9135	0.8136	0.8603	0.040*	
H11B	0.9940	0.6873	0.9079	0.040*	
C12	0.7806 (6)	0.7963 (4)	0.7168 (4)	0.0421 (11)	
H12	0.6619	0.7806	0.7371	0.051*	
C13	0.8329 (10)	0.9254 (5)	0.6618 (5)	0.078 (2)	
H13A	0.7476	0.9850	0.6514	0.094*	
H13B	0.9264	0.9450	0.6791	0.094*	
C14	0.8650 (8)	0.8592 (5)	0.5897 (4)	0.0644 (16)	
H14A	0.9779	0.8385	0.5633	0.077*	
H14B	0.7991	0.8785	0.5357	0.077*	
C15	0.9161 (5)	0.6029 (4)	0.7912 (4)	0.0315 (10)	
C16	0.8534 (5)	0.5732 (4)	0.7112 (4)	0.0375 (11)	
H16A	0.7435	0.5370	0.7513	0.045*	
H16B	0.8459	0.6471	0.6432	0.045*	
C17	0.9241 (6)	0.3704 (4)	0.7248 (4)	0.0440 (12)	
H17	0.8292	0.3407	0.7861	0.053*	
C18	1.0177 (6)	0.2925 (4)	0.6907 (4)	0.0468 (12)	
C19	0.9756 (9)	0.1617 (5)	0.7452 (6)	0.086 (2)	
H19A	0.8814	0.1425	0.8114	0.129*	
H19B	1.0675	0.1158	0.7697	0.129*	
H19C	0.9508	0.1422	0.6898	0.129*	
C20	1.1630 (6)	0.3403 (4)	0.5940 (4)	0.0430 (11)	
C21	1.1004 (5)	0.5404 (4)	0.5851 (4)	0.0376 (10)	
F1	0.6869 (3)	0.9347 (2)	0.9153 (3)	0.0508 (7)	
F2	0.1460 (4)	0.8888 (3)	0.9206 (4)	0.0897 (12)	
N1	0.5731 (5)	0.7741 (4)	1.2121 (4)	0.0512 (11)	
N2	0.4584 (5)	0.5937 (4)	1.3470 (3)	0.0525 (11)	
N3	0.6572 (4)	0.6839 (3)	1.1920 (3)	0.0322 (8)	
N4	0.8680 (4)	0.7077 (3)	0.7957 (3)	0.0298 (8)	
N5	0.9609 (4)	0.4908 (3)	0.6742 (3)	0.0351 (9)	
N6	1.1926 (4)	0.4609 (3)	0.5494 (3)	0.0411 (9)	
H6	1.2754	0.4935	0.5005	0.049*	
O1	0.7269 (3)	0.5572 (2)	1.0449 (3)	0.0351 (7)	
H1A	0.8101	0.5229	1.0622	0.053*	
O2	0.9993 (4)	0.5299 (3)	0.8517 (3)	0.0455 (8)	
O3	1.1376 (4)	0.6464 (3)	0.5414 (3)	0.0573 (10)	
O4	1.2549 (5)	0.2801 (3)	0.5518 (3)	0.0655 (11)	
C1A	0.332 (3)	0.799 (2)	0.626 (4)	0.104 (10)	0.308 (9)
H1AA	0.2307	0.7586	0.6801	0.125*	0.308 (9)
H1AB	0.3205	0.8252	0.5492	0.125*	0.308 (9)
Cl1	0.4739 (16)	0.7045 (11)	0.6375 (13)	0.193 (7)	0.308 (9)
Cl2	0.350 (3)	0.920 (2)	0.633 (2)	0.371 (15)	0.308 (9)
C1B	0.401 (4)	0.803 (2)	0.5671 (15)	0.103 (9)	0.310 (8)
H1BA	0.3007	0.7941	0.5521	0.123*	0.310 (8)
H1BB	0.4728	0.7448	0.5497	0.123*	0.310 (8)
Cl1'	0.3513 (16)	0.7336 (13)	0.7053 (12)	0.177 (6)	0.310 (8)
Cl2'	0.477 (3)	0.9237 (19)	0.453 (2)	0.340 (13)	0.310 (8)

### Atomic displacement parameters (Å^2^)

**Table:**

	*U*^11^	*U*^22^	*U*^33^	*U*^12^	*U*^13^	*U*^23^
C1	0.051 (3)	0.067 (4)	0.056 (4)	0.010 (3)	0.005 (3)	−0.036 (3)
C2	0.045 (3)	0.046 (3)	0.030 (2)	0.003 (2)	−0.003 (2)	−0.017 (2)
C3	0.028 (2)	0.041 (2)	0.032 (2)	0.0028 (18)	−0.0105 (18)	−0.019 (2)
C4	0.027 (2)	0.030 (2)	0.032 (2)	0.0003 (17)	−0.0059 (18)	−0.0146 (18)
C5	0.027 (2)	0.036 (2)	0.024 (2)	−0.0010 (18)	−0.0036 (17)	−0.0159 (18)
C6	0.027 (2)	0.034 (2)	0.043 (3)	−0.0028 (18)	−0.0070 (19)	−0.021 (2)
C7	0.047 (3)	0.038 (3)	0.059 (3)	0.010 (2)	−0.016 (2)	−0.022 (2)
C8	0.035 (3)	0.054 (3)	0.074 (4)	0.009 (2)	−0.022 (3)	−0.027 (3)
C9	0.033 (3)	0.050 (3)	0.062 (3)	−0.006 (2)	−0.015 (2)	−0.024 (3)
C10	0.033 (2)	0.037 (2)	0.034 (2)	−0.0029 (19)	−0.0070 (19)	−0.016 (2)
C11	0.025 (2)	0.044 (3)	0.038 (3)	0.0033 (18)	−0.0082 (19)	−0.026 (2)
C12	0.048 (3)	0.040 (3)	0.036 (3)	0.008 (2)	−0.012 (2)	−0.016 (2)
C13	0.137 (6)	0.041 (3)	0.054 (4)	0.002 (3)	−0.031 (4)	−0.015 (3)
C14	0.093 (4)	0.051 (3)	0.037 (3)	0.001 (3)	−0.014 (3)	−0.011 (3)
C15	0.020 (2)	0.042 (2)	0.029 (2)	0.0004 (18)	−0.0021 (18)	−0.016 (2)
C16	0.035 (2)	0.050 (3)	0.035 (2)	0.005 (2)	−0.009 (2)	−0.026 (2)
C17	0.048 (3)	0.052 (3)	0.031 (2)	−0.013 (2)	−0.005 (2)	−0.017 (2)
C18	0.058 (3)	0.043 (3)	0.044 (3)	−0.001 (2)	−0.012 (2)	−0.025 (2)
C19	0.107 (5)	0.048 (3)	0.094 (5)	−0.012 (3)	−0.007 (4)	−0.034 (4)
C20	0.050 (3)	0.051 (3)	0.046 (3)	0.012 (2)	−0.023 (2)	−0.032 (2)
C21	0.042 (3)	0.040 (3)	0.033 (2)	−0.001 (2)	−0.011 (2)	−0.017 (2)
F1	0.0455 (16)	0.0343 (14)	0.075 (2)	−0.0060 (12)	−0.0167 (14)	−0.0245 (14)
F2	0.0437 (18)	0.082 (2)	0.143 (4)	0.0242 (17)	−0.048 (2)	−0.041 (2)
N1	0.057 (3)	0.048 (2)	0.049 (3)	0.008 (2)	−0.002 (2)	−0.030 (2)
N2	0.050 (3)	0.060 (3)	0.036 (2)	−0.005 (2)	0.003 (2)	−0.019 (2)
N3	0.0328 (19)	0.043 (2)	0.0258 (19)	0.0026 (16)	−0.0049 (15)	−0.0214 (17)
N4	0.0309 (19)	0.036 (2)	0.0232 (18)	0.0019 (15)	−0.0046 (15)	−0.0153 (15)
N5	0.036 (2)	0.043 (2)	0.030 (2)	−0.0042 (16)	−0.0038 (16)	−0.0212 (17)
N6	0.039 (2)	0.050 (2)	0.033 (2)	−0.0022 (18)	−0.0007 (17)	−0.0214 (18)
O1	0.0352 (17)	0.0327 (16)	0.0405 (18)	0.0086 (13)	−0.0139 (14)	−0.0183 (14)
O2	0.0464 (19)	0.057 (2)	0.047 (2)	0.0239 (16)	−0.0270 (16)	−0.0315 (17)
O3	0.060 (2)	0.045 (2)	0.054 (2)	−0.0065 (17)	0.0030 (18)	−0.0210 (18)
O4	0.071 (3)	0.071 (2)	0.074 (3)	0.018 (2)	−0.018 (2)	−0.052 (2)
C1A	0.102 (13)	0.108 (13)	0.095 (12)	−0.001 (9)	−0.034 (9)	−0.034 (9)
Cl1	0.178 (9)	0.167 (9)	0.210 (10)	0.003 (6)	0.005 (7)	−0.096 (7)
Cl2	0.363 (17)	0.382 (18)	0.371 (18)	0.015 (10)	−0.130 (10)	−0.153 (11)
C1B	0.073 (11)	0.102 (12)	0.104 (13)	0.007 (8)	−0.023 (9)	−0.022 (9)
Cl1'	0.162 (9)	0.207 (9)	0.181 (9)	−0.023 (6)	−0.020 (6)	−0.112 (7)
Cl2'	0.310 (15)	0.319 (15)	0.367 (16)	0.024 (9)	−0.121 (10)	−0.119 (10)

### Geometric parameters (Å, °)

**Table:**

C1—N1	1.306 (6)	C15—O2	1.226 (5)
C1—N2	1.342 (6)	C15—N4	1.348 (5)
C1—H1	0.9300	C15—C16	1.519 (6)
C2—N2	1.304 (6)	C16—N5	1.460 (5)
C2—N3	1.326 (5)	C16—H16A	0.9700
C2—H2	0.9300	C16—H16B	0.9700
C3—N3	1.455 (5)	C17—C18	1.345 (6)
C3—C4	1.541 (6)	C17—N5	1.364 (6)
C3—H3A	0.9700	C17—H17	0.9300
C3—H3B	0.9700	C18—C20	1.444 (7)
C4—O1	1.418 (5)	C18—C19	1.487 (7)
C4—C5	1.517 (6)	C19—H19A	0.9600
C4—C11	1.533 (6)	C19—H19B	0.9600
C5—C10	1.383 (6)	C19—H19C	0.9600
C5—C6	1.386 (6)	C20—O4	1.222 (5)
C6—F1	1.356 (4)	C20—N6	1.362 (6)
C6—C7	1.364 (6)	C21—O3	1.211 (5)
C7—C8	1.376 (7)	C21—N6	1.366 (6)
C7—H7	0.9300	C21—N5	1.370 (5)
C8—F2	1.346 (5)	N1—N3	1.355 (5)
C8—C9	1.364 (7)	N6—H6	0.8013
C9—C10	1.378 (6)	O1—H1A	0.8200
C9—H9	0.9300	C1A—Cl1	1.580 (12)
C10—H10	0.9300	C1A—Cl2	1.581 (12)
C11—N4	1.460 (5)	C1A—H1AA	0.9600
C11—H11A	0.9700	C1A—H1AB	0.9601
C11—H11B	0.9700	C1A—H1BA	1.1262
C12—N4	1.448 (5)	Cl1—H1BB	1.0513
C12—C13	1.489 (7)	C1B—Cl1'	1.590 (12)
C12—C14	1.495 (7)	C1B—Cl2'	1.599 (12)
C12—H12	0.9800	C1B—H1AB	0.7735
C13—C14	1.488 (8)	C1B—H1BA	0.9600
C13—H13A	0.9700	C1B—H1BB	0.9601
C13—H13B	0.9700	Cl1'—H1AA	1.1355
C14—H14A	0.9700	Cl2'—H1AB	1.6877
C14—H14B	0.9700		
			
N1—C1—N2	115.8 (4)	N5—C16—C15	112.0 (3)
N1—C1—H1	122.1	N5—C16—H16A	109.2
N2—C1—H1	122.1	C15—C16—H16A	109.2
N2—C2—N3	110.8 (4)	N5—C16—H16B	109.2
N2—C2—H2	124.6	C15—C16—H16B	109.2
N3—C2—H2	124.6	H16A—C16—H16B	107.9
N3—C3—C4	111.7 (3)	C18—C17—N5	123.5 (4)
N3—C3—H3A	109.3	C18—C17—H17	118.3
C4—C3—H3A	109.3	N5—C17—H17	118.3
N3—C3—H3B	109.3	C17—C18—C20	117.6 (4)
C4—C3—H3B	109.3	C17—C18—C19	123.2 (5)
H3A—C3—H3B	107.9	C20—C18—C19	119.2 (5)
O1—C4—C5	106.0 (3)	C18—C19—H19A	109.5
O1—C4—C11	109.8 (3)	C18—C19—H19B	109.5
C5—C4—C11	112.6 (3)	H19A—C19—H19B	109.5
O1—C4—C3	109.4 (3)	C18—C19—H19C	109.5
C5—C4—C3	111.1 (3)	H19A—C19—H19C	109.5
C11—C4—C3	107.9 (3)	H19B—C19—H19C	109.5
C10—C5—C6	115.5 (4)	O4—C20—N6	120.5 (5)
C10—C5—C4	121.8 (4)	O4—C20—C18	124.1 (5)
C6—C5—C4	122.7 (4)	N6—C20—C18	115.3 (4)
F1—C6—C7	117.1 (4)	O3—C21—N6	123.1 (4)
F1—C6—C5	118.6 (4)	O3—C21—N5	122.3 (4)
C7—C6—C5	124.4 (4)	N6—C21—N5	114.5 (4)
C6—C7—C8	116.9 (4)	C1—N1—N3	101.6 (4)
C6—C7—H7	121.6	C2—N2—C1	102.3 (4)
C8—C7—H7	121.6	C2—N3—N1	109.6 (3)
F2—C8—C9	119.3 (4)	C2—N3—C3	130.1 (4)
F2—C8—C7	118.3 (4)	N1—N3—C3	120.2 (3)
C9—C8—C7	122.3 (4)	C15—N4—C12	122.2 (3)
C8—C9—C10	118.4 (4)	C15—N4—C11	118.4 (3)
C8—C9—H9	120.8	C12—N4—C11	119.2 (3)
C10—C9—H9	120.8	C17—N5—C21	121.4 (4)
C9—C10—C5	122.6 (4)	C17—N5—C16	121.9 (4)
C9—C10—H10	118.7	C21—N5—C16	116.7 (4)
C5—C10—H10	118.7	C20—N6—C21	127.5 (4)
N4—C11—C4	113.4 (3)	C20—N6—H6	121.9
N4—C11—H11A	108.9	C21—N6—H6	110.3
C4—C11—H11A	108.9	C4—O1—H1A	109.5
N4—C11—H11B	108.9	Cl1—C1A—Cl2	122 (2)
C4—C11—H11B	108.9	Cl1—C1A—H1AA	108.7
H11A—C11—H11B	107.7	Cl2—C1A—H1AA	108.8
N4—C12—C13	118.6 (4)	Cl1—C1A—H1AB	104.8
N4—C12—C14	119.8 (4)	Cl2—C1A—H1AB	104.5
C13—C12—C14	59.8 (3)	H1AA—C1A—H1AB	107.3
N4—C12—H12	115.7	Cl1—C1A—H1BA	96.4
C13—C12—H12	115.7	Cl2—C1A—H1BA	124.6
C14—C12—H12	115.7	H1AA—C1A—H1BA	92.2
C14—C13—C12	60.3 (3)	H1AB—C1A—H1BA	21.3
C14—C13—H13A	117.7	C1A—Cl1—H1BB	73.9
C12—C13—H13A	117.7	Cl1'—C1B—Cl2'	147 (2)
C14—C13—H13B	117.7	Cl1'—C1B—H1AB	106.6
C12—C13—H13B	117.7	Cl2'—C1B—H1AB	82.9
H13A—C13—H13B	114.9	Cl1'—C1B—H1BA	100.4
C13—C14—C12	59.9 (3)	Cl2'—C1B—H1BA	100.3
C13—C14—H14A	117.8	H1AB—C1B—H1BA	25.2
C12—C14—H14A	117.8	Cl1'—C1B—H1BB	99.5
C13—C14—H14B	117.8	Cl2'—C1B—H1BB	100.0
C12—C14—H14B	117.8	H1AB—C1B—H1BB	125.7
H14A—C14—H14B	114.9	H1BA—C1B—H1BB	104.4
O2—C15—N4	123.6 (4)	C1B—Cl1'—H1AA	74.3
O2—C15—C16	119.6 (4)	C1B—Cl2'—H1AB	27.0
N4—C15—C16	116.7 (4)		
			
N3—C3—C4—O1	−66.7 (4)	C17—C18—C20—N6	2.0 (7)
N3—C3—C4—C5	50.0 (4)	C19—C18—C20—N6	−179.9 (5)
N3—C3—C4—C11	173.9 (3)	N2—C1—N1—N3	−0.6 (6)
O1—C4—C5—C10	−2.4 (5)	N3—C2—N2—C1	0.6 (6)
C11—C4—C5—C10	117.7 (4)	N1—C1—N2—C2	0.1 (7)
C3—C4—C5—C10	−121.1 (4)	N2—C2—N3—N1	−1.0 (5)
O1—C4—C5—C6	176.4 (4)	N2—C2—N3—C3	−175.6 (4)
C11—C4—C5—C6	−63.5 (5)	C1—N1—N3—C2	1.0 (5)
C3—C4—C5—C6	57.7 (5)	C1—N1—N3—C3	176.2 (4)
C10—C5—C6—F1	−179.9 (4)	C4—C3—N3—C2	65.9 (6)
C4—C5—C6—F1	1.3 (6)	C4—C3—N3—N1	−108.2 (4)
C10—C5—C6—C7	0.3 (6)	O2—C15—N4—C12	175.1 (4)
C4—C5—C6—C7	−178.5 (4)	C16—C15—N4—C12	−8.5 (5)
F1—C6—C7—C8	179.4 (4)	O2—C15—N4—C11	−9.3 (6)
C5—C6—C7—C8	−0.8 (7)	C16—C15—N4—C11	167.1 (3)
C6—C7—C8—F2	179.5 (5)	C13—C12—N4—C15	−135.1 (5)
C6—C7—C8—C9	0.5 (8)	C14—C12—N4—C15	−65.5 (6)
F2—C8—C9—C10	−178.8 (5)	C13—C12—N4—C11	49.3 (6)
C7—C8—C9—C10	0.2 (8)	C14—C12—N4—C11	118.9 (5)
C8—C9—C10—C5	−0.7 (7)	C4—C11—N4—C15	−89.6 (4)
C6—C5—C10—C9	0.4 (6)	C4—C11—N4—C12	86.1 (5)
C4—C5—C10—C9	179.3 (4)	C18—C17—N5—C21	−1.3 (7)
O1—C4—C11—N4	61.5 (4)	C18—C17—N5—C16	178.0 (4)
C5—C4—C11—N4	−56.3 (5)	O3—C21—N5—C17	−177.8 (4)
C3—C4—C11—N4	−179.4 (3)	N6—C21—N5—C17	2.6 (6)
N4—C12—C13—C14	109.7 (5)	O3—C21—N5—C16	2.8 (6)
N4—C12—C14—C13	−107.7 (5)	N6—C21—N5—C16	−176.7 (4)
O2—C15—C16—N5	−27.8 (6)	C15—C16—N5—C17	100.4 (5)
N4—C15—C16—N5	155.7 (3)	C15—C16—N5—C21	−80.3 (5)
N5—C17—C18—C20	−1.1 (7)	O4—C20—N6—C21	178.6 (4)
N5—C17—C18—C19	−179.2 (5)	C18—C20—N6—C21	−0.6 (7)
C17—C18—C20—O4	−177.2 (5)	O3—C21—N6—C20	178.8 (5)
C19—C18—C20—O4	0.9 (8)	N5—C21—N6—C20	−1.7 (6)

### Hydrogen-bond geometry (Å, °)

**Table:**

*D*—H···*A*	*D*—H	H···*A*	*D*···*A*	*D*—H···*A*
N6—H6···N2^i^	0.80	2.11	2.893 (5)	167.
O1—H1A···O2^ii^	0.82	2.13	2.903 (4)	158.

Symmetry codes: (i) *x*+1, *y*, *z*−1; (ii) −*x*+2, −*y*+1, −*z*+2.
